# Elastic and Thermoelastic Responses of Orthotropic Half-Planes

**DOI:** 10.3390/ma15010297

**Published:** 2021-12-31

**Authors:** Yuriy V. Tokovyy, Anatoliy V. Yasinskyy, Sebastian Lubowicki, Dariusz M. Perkowski

**Affiliations:** 1 Pidstryhach Institute for Applied Problems of Mechanics and Mathematics, National Academy of Sciences of Ukraine, 79060 Lviv, Ukraine; tokovyy@gmail.com (Y.V.T.); yasinskyy.anatoliy@gmail.com (A.V.Y.); 2 Department of Applied Mathematics, Institute of Applied Mathematics and Fundamental Sciences, National University Lviv Polytechnic, 79013 Lviv, Ukraine; 3 Faculty of Mechanical Engineering, Bialystok University of Technology, 15-351 Bialystok, Poland; s.lubowicki@doktoranci.pb.edu.pl

**Keywords:** orthotropic material, elastic half-plane, plane elasticity and thermoelasticity problems, explicit solutions, integral equation of second kind, resolvent-kernel algorithm

## Abstract

A unified approach is presented for constructing explicit solutions to the plane elasticity and thermoelasticity problems for orthotropic half-planes. The solutions are constructed in forms which decrease the distance from the loaded segments of the boundary for any feasible relationship between the elastic moduli of orthotropic materials. For the construction, the direct integration method was employed to reduce the formulated problems to a governing equation for a key function. In turn, the governing equation was reduced to an integral equation of the second kind, whose explicit analytical solution was constructed by using the resolvent-kernel algorithm.

## 1. Introduction

The design of engineering applications and advancement of material science are driven projection and implementation of new composite materials with averaged properties meeting specific requirements [1]. These requirements are usually formulated through both empirical evidence and a priori analysis implemented by inverse and optimization algorithms [2,3,4]. Such algorithms, in turn, are based on the complex analysis of primary responses of multi-phase materials to the collective action of physical impacts, accounting for a wide range of operational and constructional features [5,6] (e.g., anisotropy of materials, fiber packing, and stratification). Moreover, the efficiency in developing optimization algorithms and procedures for the inverse identification of effective material parameters for advanced composite materials are directly related to the solutions of problems in solid mechanics [7,8,9].

A homogenization-based approach is widely used to predict the effective mechanical or thermal properties in solid mechanics problems. Due to the large number of layers forming, for instance, a stratified composite, and the need to meet the continuity conditions for the temperature and the components of displacement, stress, and heat-flux vectors, the implementation of approaches based on the classical theory of thermoelasticity becomes problematic regarding the related boundary value problems for composite materials. Therefore, it is necessary to use specific approximate models that meet the original interface conditions on the surfaces connecting the individual components. These models are created by applying various averaging procedures, e.g., the thick plate theory approach [10], mixture theory approach [11], asymptotic homogenization [12], matrix methods [13], and tolerance theory [14]. Another approach is homogenization based on non-asymptotic techniques derived from non-standard analysis, which was proposed in [15] and adapted for composites of layered structure [16]. Within the framework of this approach, the homogenized model with microlocal parameters is now widely used in composite materials research [17,18]. The new approach to this model has also found application in modeling layered composites with functionally graded properties for thermoelastic problems [19]. 

An interesting approach for handling composite materials consisting of a base layer covered with thin multilayer coatings of multifunctional purpose is based on the method of generalized boundary condition [20,21]. This approach captures the effect of thin multilayer coating and avoids computational complications due to the mismatch of the geometrical and thermophysical properties on the layers’ interfaces.

Efficient analysis of the elastic and thermoelastic response of composite materials exhibiting anisotropic features significantly depends on the accuracy of accounting for the dissimilarity of the material moduli in different spatial directions [22,23,24]. Hence, the methods for such analysis of isotropic solids may largely fail when attempted directly on anisotropic solids. If, for example, the eigenfunction method was used (separation of variables) to studying local effects in semi-infinite elastic composites, the desired solution is to decay when moving away from the loaded area according to the Saint-Venant principle. In contrast to the isotropic case, however, the eigenvalues of such problems for anisotropic solids will no longer be defined uniquely but depend on the interrelation between the elastic moduli involved into the coefficients of characteristic equations [6,25]. This affects the type and character of corresponding eigenfunctions and complicates them, ensuring their vanishing behavior at distant points. The latter drawback limits the applicability of such solutions for the entire spectra of feasible anisotropic moduli. 

In this paper, we extend a technique that avoids the latter complication to represent a solution for an elastic orthotropic half-plane in a unified form that explicitly depends on the locally applied loadings and ensures decaying at a distance from the loaded zone. The technique is based on applying the direct integration method [6], which implies the reduction of a corresponding boundary value problem to the governing equations for individual stress tensor components by using the relationships derived via the integration of the equilibrium equations. This method has been efficiently used to analyze numerous direct and inverse boundary value problems in different coordinate systems [26,27]. In [28], this method was used to develop a technique for solving a three-dimensional elasticity problem for a transversely isotropic half-space. This technique reduces governing equations of the problem to integral equations of the second kind. With the application of the resolvent-kernel algorithm for solving the derived integral equations, solutions are constructed in forms that are irrespective of the interdependences between the elastic material moduli and decays on the distance from the loaded zones. Moreover, the solution is given in the form of explicit expressions on the loadings. These features make it quite attractive for the identification, inverse, and optimization algorithms [29].

## 2. Formulation of the Problem

Consider a plane thermoelasticity problem for an elastic orthotropic half-plane (x,y)∈(−∞,∞)×[0,∞) in the dimensionless Cartesian coordinate system (x,y). The problem is governed [22,30] by the equations of equilibrium in terms of stresses

(1) $$\frac{\partial \sigma_{xx}}{\partial x}+\frac{\partial \sigma_{xy}}{\partial y}=0,\;\frac{\partial \sigma_{xy}}{\partial x}+\frac{\partial \sigma_{yy}}{\partial y}=0$$

and the strain-compatibility equation

(2) $$\frac{\partial^{2}\varepsilon_{xx}}{\partial y^{2}}+\frac{\partial^{2}\varepsilon_{yy}}{\partial x^{2}}=\frac{\partial^{2}\varepsilon_{xy}}{\partial x\partial y}$$

Here, $\sigma_{xx}$, $\sigma_{yy}$, $\sigma_{xy}$ and $\varepsilon_{xx}$, $\varepsilon_{yy}$, $\varepsilon_{xy}$ are the in-plane normal and tangential components of the stress- and strain-tensor, respectively. These components are related via the constitutive equations of the Duhamel–Neumann law for orthotropic materials [31], which takes the following form

(3) $$\varepsilon_{xx}=a_{11}\sigma_{xx}+a_{12}\sigma_{yy}+\alpha_{1}T,\;\varepsilon_{yy}=a_{12}\sigma_{xx}+a_{22}\sigma_{yy}+\alpha_{2}T,\;G_{xy}\varepsilon_{xy}=\sigma_{xy}$$

under both the plane strain and plane stress hypotheses. Here,

(4) $$\begin{array}{c} a_{11}=\frac{{\tilde{a}}_{11}}{E_{x}},\;a_{22}=\frac{{\tilde{a}}_{22}}{E_{y}},\;a_{12}=-\frac{{\tilde{a}}_{12}}{E_{y}}=-\frac{{\tilde{a}}_{21}}{E_{x}},\\ {\tilde{a}}_{11}=\left\{\begin{array}{l} 1,\\ 1-\nu_{zx}\nu_{xz}, \end{array}\right.\;{\tilde{a}}_{22}=\left\{\begin{array}{l} 1,\\ 1-\nu_{zy}\nu_{yz}, \end{array}\right.\\ {\tilde{a}}_{12}=\left\{\begin{array}{l} \nu_{yx},\\ \nu_{yx}+\nu_{zx}\nu_{yz}, \end{array}\right.\;{\tilde{a}}_{21}=\left\{\begin{array}{l} \nu_{xy},\\ \nu_{xy}+\nu_{xz}\nu_{zy}, \end{array}\right.\\ \alpha_{1}=\left\{\begin{array}{l} \alpha_{x},\\ \alpha_{x}+\alpha_{z}\nu_{zx}, \end{array}\right.\;\alpha_{2}=\left\{\begin{array}{l} \alpha_{y},\\ \alpha_{y}+\alpha_{z}\nu_{zy}, \end{array}\right. \end{array}$$

$E_{x}$ and $E_{y}$ are the Young moduli in the directions x and y, respectively; $\nu_{jl}$ are the Poisson ratios describing the contraction in the l-direction at tension in the *j*-direction, l,j={x,y,z}, l≠j; $G_{xy}$ is the shear modulus for the coordinate plane (x,y); $\alpha_{x},$ $\alpha_{y}$, and $\alpha_{z}$ are the linear thermal expansion coefficients in the directions x,y, and z, respectively; $T(x,y)=T^{*}(x,y)-T_{0}^{*}(x,y)$, $T^{*}(x,y)$ is the temperature field, which can be found from a corresponding heat conduction problem [31], and $T_{0}^{*}(x,y)$ is the reference temperature of the non-stressed state. In the foregoing expressions with braces, the upper and lower lines correspond to the plane stress and plane strain cases, respectively. The orthotropic elastic modules in (4) comply with the following symmetry condition [22]

$$E_{j}\nu_{lj}=E_{l}\nu_{jl},\;l,j=\{x,y,z\},\;\;\;l\neq j.$$

Assume boundary y=0 of the half-plane to be acted upon by locally distributed normal and tangential force loadings imposed by the following boundary conditions:

(5) $$\sigma_{yy}=-p(x),\;\sigma_{xy}=q(x),\;\;y=0,\;|x|\;<\infty ,$$

where p(x) and q(x) are the given functions of some local distribution profiles, i.e., $\underset{|x|\;\to \;\infty }{\lim}p(x)=$$\underset{|x|\;\to \;\infty }{\lim}q(x)=0$. Note that for the correct formulation of the boundary value problem, the force loadings (5) and temperature T(x,y) are to meet the necessary conditions derived in [32].

## 3. Reduction to Governing Equations

By following the strategy of the direct integration method [6,26], consider the normal stress $\sigma_{yy}$ and the in-plane total stress $\sigma =\sigma_{xx}+\sigma_{yy}$ to be the key functions. By eliminating the tangential stress from equilibrium Equation (1), we derive the following governing equation for the key functions:

(6) $$\Delta \sigma_{yy}=\frac{\partial^{2}\sigma }{\partial x^{2}}$$

where $\Delta =\partial^{2}/\partial x^{2}+\partial^{2}/\partial y^{2}$ is the in-plane Laplace differential operator. This equation implies general equilibrium in terms of the stress-tensor components and thus is irrespective of the material properties.

Making use of the constitutive Equation (3) along with the equilibrium ones (1), we can represent the compatibility Equation (2) in terms of stresses, as follows:

(7) $$\Delta \langle a_{11}\sigma +\alpha_{1}T\rangle =(\alpha_{1}-\alpha_{2})\frac{\partial^{2}T}{\partial x^{2}}+\left(2\beta_{1}-\frac{1}{G_{xy}}\right)\frac{\partial^{2}\sigma_{yy}}{\partial y^{2}}-\beta_{2}\frac{\partial^{2}\sigma_{yy}}{\partial x^{2}}$$

Here, $\beta_{1}=a_{11}-a_{12},$ $\beta_{2}=a_{22}-a_{11}$. Within the context of expressions (4), the coefficients in governing Equation (7) involve the orthotropic moduli of the material. Note that this equation can also be obtained by assuming the material moduli constant in an analogous equation [33] derived for inhomogeneous orthotropic material. In the case of isotropic material, Equation (7) can naturally be reduced to the corresponding governing equation derived in [32].

When acting upon Equation (7) by the differential operator $\partial^{2}/\partial x^{2}$ and making use of Equation (6), we can derive the following fourth-order differential equation:

(8) $$\frac{\partial^{4}\sigma_{yy}}{\partial y^{4}}+2a_{1}\frac{\partial^{4}\sigma_{yy}}{\partial x^{2}\partial y^{2}}+a_{2}\frac{\partial^{4}\sigma_{yy}}{\partial x^{4}}=-\frac{\partial^{2}}{\partial x^{2}}\left(\alpha_{1}^{0}\frac{\partial^{2}T}{\partial y^{2}}+\alpha_{2}^{0}\frac{\partial^{2}T}{\partial x^{2}}\right)$$

for the stress $\sigma_{yy}$, solely, where the coefficients are given as

(9) $$a_{1}=\frac{1}{a_{11}}\left(a_{12}+\frac{1}{2G_{xy}}\right),\;\;\;\;\;a_{2}=\frac{a_{22}}{a_{11}},\;\;\;\;\;\;\;\;\alpha_{j}^{0}=\frac{\alpha_{j}}{a_{11}},\;\;\;\;\;\;j=1,2$$

By using the second equilibrium Equation (1) together with the second boundary condition (5), we can derive a condition for the derivative of the key function in the form as follows:

(10) $$\frac{\partial \sigma_{yy}}{\partial y}=-\frac{dq(x)}{dx},\;\;y=0,\;|x|\;<\infty .$$

In such a manner, the original thermoelasticity problem is reduced to a governing Equation (8) with coefficients (9) expressed through the orthotropic elastic moduli (4) under the boundary conditions presented by the first condition (5) and condition (10) for the key function $\sigma_{yy}$. After this function is found by solving the mentioned boundary value problem, the in-plane total stress σ can be determined from Equation (6). The normal stress $\sigma_{xx}$ can then be found via the formula $\sigma_{xx}=\sigma -\sigma_{yy}$. The tangential stress $\sigma_{xy}$ can be resulted as follows

$$\sigma_{xy}=\frac{q(x)}{2}-\frac{1}{2}\int_{0}^{\infty }\frac{\partial \sigma_{xx}(x,\eta )}{\partial x}\mathrm{sgn}(y-\eta )d\eta =-\frac{1}{2}\int_{-\infty }^{\infty }\frac{\partial \sigma_{yy}(\xi ,y)}{\partial y}\mathrm{sgn}(x-\xi )d\xi$$

based on the integration of the equilibrium Equation (1) within the context of boundary condition in (5).

## 4. Solution of the Governing Equations

To solve the formulated problem, we employ the integral Fourier transform with respect to variable x:

(11) $$\bar{f}(y)=\bar{f}(y;s)=\int_{-\infty }^{\infty }f(x,y)\exp(-isx)dx$$

where s is the transform parameter, $i^{2}=-1$, and f(x,y) is an arbitrary function whose transform (11) exists in at least the generalized sense [34].

Having applied transform (11) to Equations (8) and (10) along with the first condition in (5), we arrive at the following ordinary differential equation:

(12) $$\frac{d^{4}{\bar{\sigma }}_{yy}}{dy^{4}}-2s^{2}a_{1}\frac{d^{2}{\bar{\sigma }}_{yy}}{dy^{2}}+s^{4}a_{2}{\bar{\sigma }}_{yy}=s^{2}\left(\alpha_{1}^{0}\frac{d^{2}\bar{T}}{dy^{2}}-s^{2}\alpha_{2}^{0}\bar{T}\right)$$

accompanied with the conditions

(13) $${\bar{\sigma }}_{yy}=-\bar{p},\;\frac{d{\bar{\sigma }}_{yy}}{dy}=-is\bar{q},\;\;y=0.$$

The characteristic equation of (12) takes the following form

(14) $$\mu^{4}-2a_{1}{\left(s\mu \right)}^{2}+s^{4}a_{2}=0$$

with μ being the eigenvalues, which can be given as

(15) μj=(−1)jλ1s,  μ2+j=(−1)jλ2s,    j=1,2,  λ1=a1−a12−a2=λ1Re+iλ1Im,  λ2=a1+a12−a2=λ2Re+iλ2Im.

As it can be seen from formulae in (15), the type of the eigenvalues critically depends on the interrelation between the coefficients $a_{1}$ and $a_{2}$, which is verbalized by expressions for $\lambda_{1}$ and $\lambda_{2}$. Thus, in order to be able of capturing all possible features of the solution to Equation (12) for various orthotropic materials and ensuring its vanishing with y→∞, it is necessary to exhaustively analyze all possible cases of interrelation between coefficients of Equation (14), which within the context of formules (4) and (9), implies the analysis of interdependences of the orthotropic material moduli. Based on the theoretical and experimental studies [22,35], the following constraints can be established for the elastic moduli involved into expressions for the coefficients (9):

(16) $$\begin{array}{c} E_{l}>0,\;G_{12}>0,\;l=1,2,3,\;|\nu_{13}+\nu_{12}\nu_{23}|\;<\frac{1}{\sqrt{E_{2}E_{3}}}\sqrt{E_{1}-\nu_{12}E_{2}}\sqrt{E_{2}-\nu_{23}E_{3}},\\ |\nu_{12}|\;<\sqrt{\frac{E_{1}}{E_{2}}},\;|\nu_{21}|\;<\sqrt{\frac{E_{2}}{E_{1}}},\;|\nu_{23}|\;<\sqrt{\frac{E_{2}}{E_{3}}},\;|\nu_{32}|\;<\sqrt{\frac{E_{3}}{E_{2}}}. \end{array}$$

In view of the equations in (4), (9), and (16), it can be concluded that

$$a_{2}>0$$

**Figure 1:**
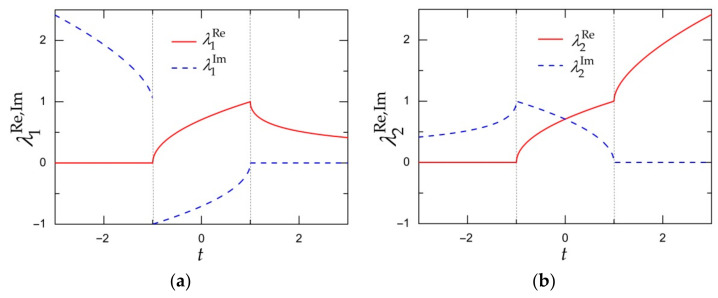
The real and imaginary parts of eigenvalues $\lambda_{1}$ (**a**) and $\lambda_{2}$ (**b**) in formulae (15) versus $t=a_{1}/\sqrt{a_{2}}$ under constraints (16).

The feasible dependences (i.e., the ones satisfying conditions (16)) of the real and imaginary parts λjRe/a24 and λjIm/a24, j=1,2, of eigenvalues (15) on parameter $t=a_{1}/\sqrt{a_{2}}$ are shown in Figure 1. The corresponding expressions for λjRe/a24 and λjIm/a24 are given in Table A1 of Appendix A.

The observed variation in the type and multiplicity of the eigenvalues for all feasible dispersions of the orthotropic material moduli affects significantly the form of a general solution to Equation (12), which is critical for assuring its vanishing when moving away from the loaded boundary y=0 of the half-plane towards its depth. To the best of our knowledge, the exhaustive coverage of all possible cases of vanishing analytical solutions remained beyond the circle of interest in the vast majority of papers dedicated to the mechanical analysis of semi-infinite orthotropic solids.

The cumbersomeness of the exhaustive analyzes of all possible cases of interdependences between the anisotropic material moduli was demonstrated in [25] when solving a three-dimensional problem in a half-space made of a relatively simpler type of material, i.e., a transversely isotropic one, possessing a smaller number of independent elastic moduli compared to an orthotropic material. In order to avoid this cumbersomeness, it was suggested in [28] to use an alternative way for constructing analytical solutions to such problems by reducing them to integral equations of the second kind with subsequent application of the resolvent-kernel method. Herein, we extend the latter technique for solving the boundary value problem (12) and (13) for the case of orthotropic material. 

By following the strategy of paper [28], let us represent Equation (12) in the form as follows:

(17) $$\frac{d^{4}{\bar{\sigma }}_{yy}}{dy^{4}}-2s^{2}a^{2}\frac{d^{2}{\bar{\sigma }}_{yy}}{dy^{2}}+s^{4}a^{4}{\bar{\sigma }}_{yy}=2s^{2}(a_{1}-a^{2})\frac{d^{2}{\bar{\sigma }}_{yy}}{dy^{2}}+s^{2}\left(\alpha_{1}^{0}\frac{d^{2}\bar{T}}{dy^{2}}-s^{2}\alpha_{2}^{0}\bar{T}\right),$$

where $a^{4}=a_{2}>0$. A general solution to Equation (17) that is vanishing at y→∞ can be given as

(18) $${\bar{\sigma }}_{yy}(y)=(A_{1}+yA_{2})\exp(-|s|ay)\;-\frac{\left|s\right|}{2}\frac{a_{1}-a^{2}}{a}\int_{0}^{\infty }{\bar{\sigma }}_{yy}(\eta )\left(1-|s|a|y-\eta |\right)\exp(-|s|a|y-\eta |)d\eta \;-\frac{\left|s\right|}{4a^{3}}\int_{0}^{\infty }\bar{T}(\eta )(\left(1+|s|a|y-\eta |\right)\alpha_{2}^{0}+a^{2}\left(1-|s|a|y-\eta |\right)\alpha_{1}^{0})\exp(-|s|a|y-\eta |)d\eta ,$$

where $A_{j}$, j=1,2, are constants of integration. These constants can be determined by putting (18) into conditions (13). As a result, we arrive at the following equation for the key stress ${\bar{\sigma }}_{yy}$:

(19) $${\bar{\sigma }}_{yy}(y)=-\;(\left(1+|s|ay\right)\bar{p}+isy\bar{q})\exp(-|s|ay)+\Theta (y)+\int_{0}^{\infty }{\bar{\sigma }}_{yy}(\eta )K(y,\eta )d\eta$$

Here,

$$K(y,\eta )=\frac{\left|s\right|}{2}\frac{a_{1}-a^{2}}{a}(\left(1-|s|a\eta +|s|ay(3-2|s|a\eta )\right)\exp(-|s|a(y+\eta ))$$

$$-\;\left(1-|s|a|y-\eta |\right)\exp(-|s|a|y-\eta |))$$

$$\Theta (y)=\frac{\left|s\right|}{4a^{3}}\int_{0}^{\infty }\bar{T}(\eta )(\left\{\left(1\;+\;|s|a\eta \;+|s|ay(1+2|s|a\eta )\right)\alpha_{2}^{0}\right.$$

$$\left.+\;a^{2}\left(1-|s|a\eta +|s|ay(3-2|s|a\eta )\right)\alpha_{1}^{0}\right\}\exp(-|s|a(y+\eta ))$$

$$-\left\{\left(1+|s|a|y-\eta |\right)\alpha_{2}^{0}+a^{2}\left(1-|s|a|y-\eta |\right)\alpha_{1}^{0}\right\}\exp(-|s|a|y-\eta |))d\eta$$

Equation (19) is an integral equation of the second kind. An analytical solution to this equation expressing the key function ${\bar{\sigma }}_{yy}(y)$ through the mapping functions of the force loadings $\bar{p}$ and $\bar{q}$ on the boundary and the temperature $\bar{T}(y)$, can be constructed by means of the resolvent-kernel algorithm [36] to obtain the following:

(20) $${\bar{\sigma }}_{yy}(y)=-\bar{p}\varphi_{p}(y)-is\bar{q}\varphi_{q}(y)+\varphi_{T}(y)$$

where

$$\varphi_{p}(y)=\left(1+|s|ay\right)\exp(-|s|ay)+\int_{0}^{\infty }\left(1+|s|a\eta \right)R(y,\eta )\exp(-|s|a\eta )d\eta$$

$$\varphi_{q}(y)=y\exp(-|s|ay)+\int_{0}^{\infty }\eta R(y,\eta )\exp(-|s|a\eta )d\eta$$

$$\varphi_{T}(y)=\Theta (y)+\int_{0}^{\infty }\Theta (\eta )R(y,\eta )d\eta$$

and the resolvent kernel is constructed as

(21) $$R(y,\eta )=\sum_{n=0}^{\infty }K_{n+1}(y,\eta )$$

Here,

$$K_{1}(y,\eta )=K(y,\eta ),\;\;K_{n+1}(y,\eta )=\int_{0}^{\infty }K_{1}(y,\tau )K_{n}(\tau ,\eta )d\tau ,\;n=1,2,\ldots$$

Note that for many practical cases, the resolvent kernel (21) can be found in a closed analytical form [36]. Otherwise, the practical computation of the resolvent can be implemented using the approximate formula

(22) $$R(y,\eta )\approx R_{N}(y,\eta )=\sum_{n=0}^{N}K_{n+1}(y,\eta )$$

where N is an integer, which can be evaluated either by a numerical experiment or through an analytical procedure [37].

After constructing the stress ${\bar{\sigma }}_{yy}(y)$ in the form (20), we can use the following formulae:

(23) $${\bar{\sigma }}_{xx}(y)=-\frac{1}{s^{2}}\frac{d^{2}{\bar{\sigma }}_{yy}(y)}{dy^{2}},\;\;\;{\bar{\sigma }}_{xy}(y)=\frac{i}{s}\frac{d{\bar{\sigma }}_{yy}(y)}{dy},$$

derived from Equation (1) in the mapping domain of the integral transform (11). By substituting (20) into (23), we can represent the stresses ${\bar{\sigma }}_{xx}(y)$ and ${\bar{\sigma }}_{xy}(y)$ in a similar to (20) form, as follows:

(24) $${\bar{\sigma }}_{xx}(y)=-\bar{p}\Psi_{p}(y)-is\bar{q}\Psi_{q}(y)+\Psi_{T}(y)$$

(25) $${\bar{\sigma }}_{xy}(y)=-\bar{p}\vartheta_{p}(y)-is\bar{q}\vartheta_{q}(y)+\vartheta_{T}(y)$$

The expressions for functions $\Psi_{p}(y)$, $\Psi_{q}(y)$, $\Psi_{T}(y)$, and $\vartheta_{p}(y)$, $\vartheta_{q}(y)$, and $\vartheta_{T}(y)$ are given in Appendix B.

After constructing all the stress-tensor components in the mapping domain of the transform (11) in the form (20), (24), and (25), we can restore them in the physical domain by implementing the inverse transform

$$f(x,y)=\frac{1}{2\pi }\int_{-\infty }^{\infty }\bar{f}(y;s)\exp(isx)ds$$

The constructed expressions (20), (24), and (25) allow for expressing the stress tensor components in an orthotropic half-plane through the applied force loadings and the temperature field explicitly. They appear to be uniform for any feasible case of material properties obeying the constraints (16) and ensuring the stress tensor components exhibit decaying behavior at the infinitely distant points of the half-plane.

## 5. Numerical Example and Discussion

Consider numerical implementation of the proposed solution algorithm for several case studies of the orthotropic materials with properties presented in Table 1 for the case of plane stress adopted in expressions for elastic coefficients in (4). The considered orthotropic composite materials are based on carbon fiber plastics which are of practical implementation for aircraft engineering. The computations are performed for the smooth normal impact of the half-plane boundary (5), where $p(x)=p_{0}\exp(-\kappa x^{2})$ and q(x)=0, $p_{0}=\mathrm{const}$, κ=const. It can be shown that the eigenvalues $\lambda_{1}$ and $\lambda_{2}$ in formulae (15) are complex conjugated for material *1* and real and dissimilar for material *2* from Table 1.

**Figure 2:**
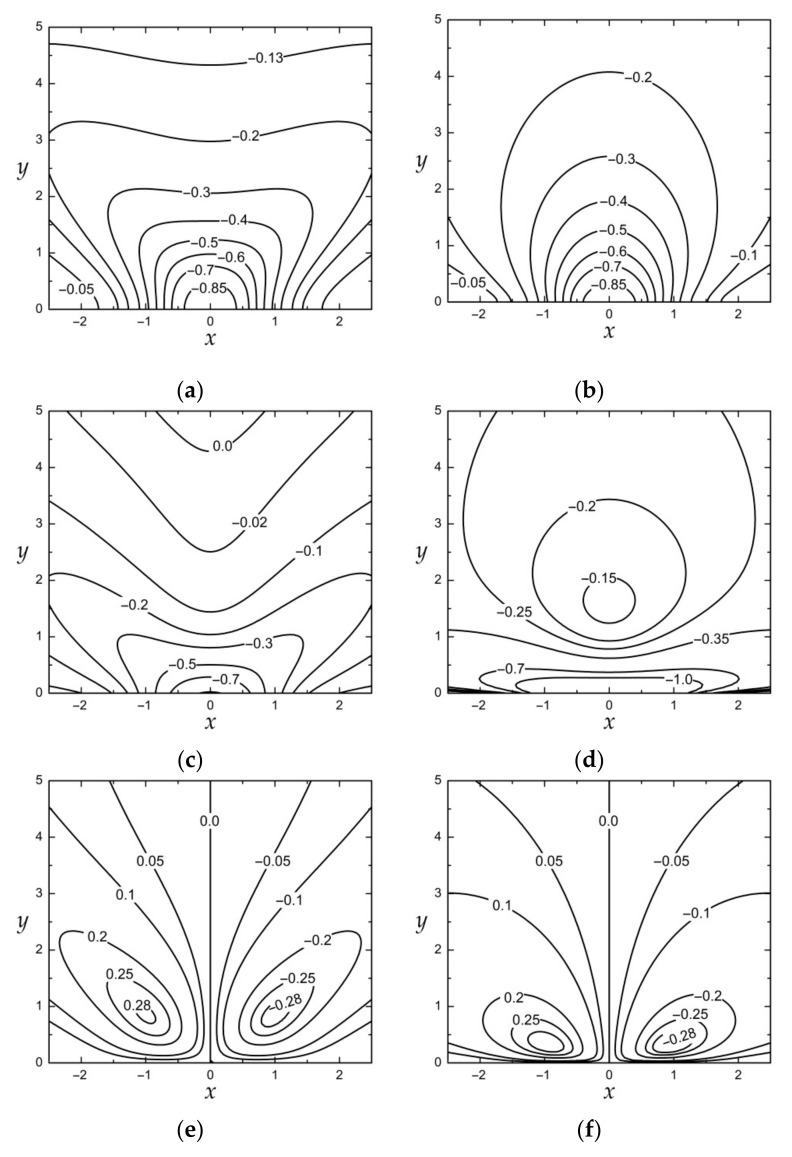
The full-field distributions of the dimensionless stress tensor components for the orthotropic composite materials with properties given in Table 1 under the normal force loading $p(x)=p_{0}\exp(-\kappa x^{2})$ at κ=1 : (**a**) $\sigma_{yy}/p_{0}$ for material *1*, (**b**) $\sigma_{yy}/p_{0}$ for material *2*, (**c**) $\sigma_{xx}/p_{0}$ for material *1*, (**d**) $\sigma_{xx}/p_{0}$ for material *2*, (**e**) $\sigma_{xy}/p_{0}$ for material *1*, (**f**) $\sigma_{xy}/p_{0}$ for material *2*.

The full-field distributions of the dimensionless stress tensor components $\sigma_{yy}/p_{0}$, $\sigma_{xx}/p_{0}$, and $\sigma_{xy}/p_{0}$ are depicted in Figure 2 for material *1* (a, c, e) and *2* (b, d, f) demonstrating the effect of anisotropy in the stress patterns for the considered type of loading. The computations have been performed by making use of the exact solutions to Equation (12).

**Figure 3:**
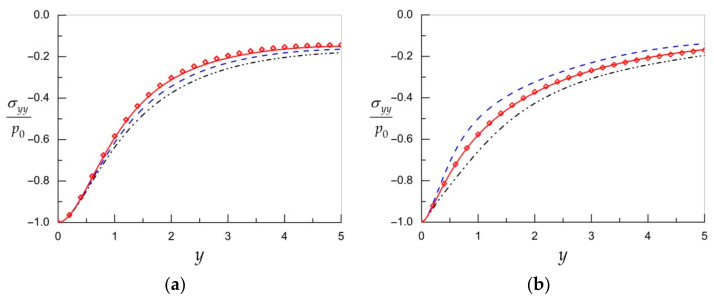
The dimensionless stress $\sigma_{yy}/p_{0}$ at x=0 and κ=1 for material *1* (**a**) and material *2* (**b**) under different values of N in formula for resolvent kernel (22): N=1 (dash-dot lines), N=2 (dash lines), N=3 (solid lines), and exact solutions (diamonds).

Figure 3 demonstrates the dimensionless stress $\sigma_{yy}/p_{0}$ computed at x=0 and κ=1 by unified formula (20) for different values of N in the expression for approximate resolvent kernel (22) used instead of the exact one (21) for the orthotropic materials with properties given in Table 1. As we can see, keeping three terms in the series approximating the resolvent kernel is enough for achieving engineering accuracy. The approximation towards the exact solution depends on the character of the eigenvalues. For the case of complex-conjugated eigenvalues (material *1*), the succession is single sided, while it is flipping from side to side for the case of real eigenvalues (material *2*).

Consider the application of the developed solution technique for the computation of thermal stresses in orthotropic half-planes in the case of plane strain (bottom lines under the braces in expressions (4)) for the materials with properties given in Table 2. The analysis of the case of plane strain involves a greater number of material moduli compared to the plane-stress case considered above.

Assuming the heat conduction moduli to be the same in all the spatial directions, let the thermal stresses be induced by the steady-state temperature field, with the Fourier mapping (11) given in the form

(26) $$\bar{T}(y)=-s^{2}\frac{\tau_{0}\sqrt{\pi }}{4}\exp\left(-|s|y-\frac{s^{2}}{4}\right),\;\;\;\tau_{0}=\mathrm{const}$$

This temperature field was derived and discussed in detail in [32], emphasizing it fits the correctness conditions for the solutions to stationary thermoelasticity problems in half-planes [6,32]. For this case study, the boundary of the half-plane is assumed to be free of external force loadings, i.e., p(x)=q(x)=0 in (5). 

**Figure 4:**
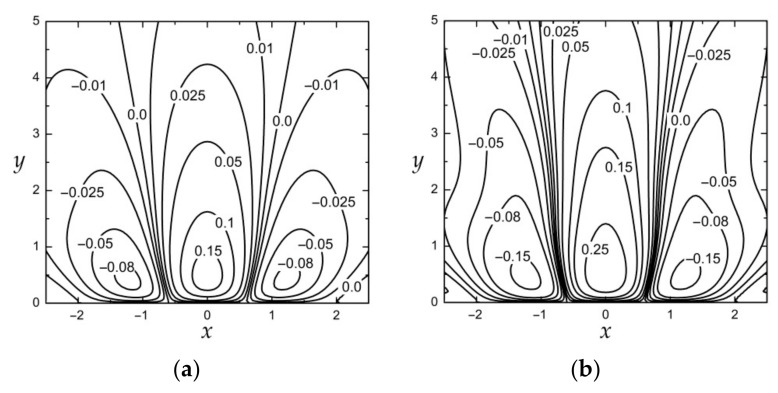
The full-field distributions of the thermal stress $\sigma_{yy}$ [GPa] for orthotropic composite materials with properties given in Table 2 due to the temperature field with Fourier image (26) at $\tau_{0}=1$ : (**a**) material *3*, (**b**) material *4*.

The effect of material anisotropy in the thermal stresses is illustrated in Figure 4 depicting the full-field distributions of the stress $\sigma_{yy}$ for materials 3 and 4 (see Table 2). Note that eigenvalues (15) for both materials are real and dissimilar. We can observe the similarity in the character of stress distribution for the considered orthotropic materials. However, the magnitude of thermal stresses is significantly different.

**Figure 5:**
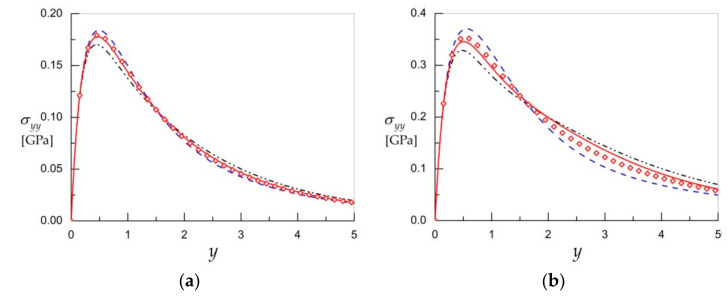
The thermal stress $\sigma_{yy}$ at x=0 due to thermal field (26) for material *3* (**a**) and material *4* (**b**) under different values of N in formula for resolvent kernel (22): N=1 (dash–dot lines), N=2 (dash lines), N=3 (solid lines), and exact solutions (diamonds).

The convergence of solutions when implementing the approximate formula for the resolvent kernel (22) instead of the exact one for the computation of thermal stress (20) is demonstrated in Figure 5. Similar to the preceding case studies for the half-plane subject to normal force loadings, we can observe good convergence of the approximate solution to the exact one, i.e., keeping three terms in the series presenting the resolvent kernel is sufficient for achieving the desired accuracy. For material 4 (Figure 5b)*,* the convergence is slightly slower.

## 6. Conclusions

Advanced design and processing of machinery, equipment, and structures elements are associated with the development of technologies for creating composite materials with properties optimized by specific target criteria. This encourages complex studies of physicochemical processes and thermal stresses affecting the strength and durability of materials and structures under the complex action of the dominant types of loading, such as the force and temperature fields, taking into account a wide range of operational and structural features. 

A technique for solving the plane elasticity and thermoelasticity problems for an orthotropic homogeneous half-plane is developed based on the direct integration method. The key features of the presented technique are the following.

- This technique allows for constructing an analytical solution in the form that is unified for all possible interdependencies between the elastic moduli of an orthotropic material.
- For all possible cases of orthotropic materials, the solution fits the Saint-Venant principle, i.e., decreases when receding the loaded zone of the half-plane boundary.
- The solution is constructed in the form of explicit expressions of the stress tensor components on the applied force and thermal loadings.

These features make the constructed solutions to be a perfect candidate for implementation with the engineering analysis within the inverse problems, optimization of the stress patterns, identification of effective material parameters, etc.

As a certain disadvantage of the proposed solution scheme, it can be noted that the practical computation of the solution involves approximated formula (22) for the resolvent kernel. Nevertheless, the considered case studies have shown rapid convergence of the approximate solution to the exact one.

## Figures and Tables

**Table 1:** Material properties of some orthotropic composites [38].

	Material	$E_{x},\;\mathrm{GPa}$	$E_{y},\;\mathrm{GPa}$	$\nu_{xy}$	$G_{xy},\;\mathrm{GPa}$
*1*	Carbon Fiber Composite UP-1	15.70	14.80	0.67	42.00
*2*	Carbon Fiber Textile Reinforced Plastic UOL-300-1A	150.70	8.00	0.29	4.00

**Table 2:** Elastic and thermoelastic material moduli of some orthotropic composites.

	Material	$E_{x},\;\mathrm{GPa}$	$E_{y},\;\mathrm{GPa}$	$\nu_{yx}$	$\nu_{zx}$	$\nu_{zy}$	$G_{xy},\;\mathrm{GPa}$	$\alpha_{x}\text{×}\;10^{5},\;1/K$	$\alpha_{y}\text{×}\;10^{5},\;1/K$	$\alpha_{z}\text{×}\;10^{5},\;1/K$
*3*	Glass-Epoxy Composite [39]	13.7	55.9	0.277	0.4	0.68	5.6	4.0	1.0	4.0
*4*	Boron-Epoxy Fiber-Reinforced Composite [40]	2.068	20.68	0.25	0.25	0.25	1.034	5.1	0.84	5.1

## Data Availability

The data presented in this study are available on request from the corresponding author.

## Appendix A. Eigenvalues of the Characteristic Equation

**Table A1:** Expressions for the real and imaginary parts λjRe/a24 and λjIm/a24, j=1,2, of eigenvalues (15) on parameter $t=a_{1}/\sqrt{a_{2}}$ for the material moduli satisfying physical constraints (16).

$t=a_{1}/\sqrt{a_{2}}$	λ1Re/a24	λ1Im/a24	λ2Re/a24	λ2Im/a24
t<−1	0	0	$\sqrt{|t|+\sqrt{t^{2}-1}}$	$\sqrt{|t|-\sqrt{t^{2}-1}}$
t=−1	0	0	1	1
−1<t<0	$\sin\left(\frac{1}{2}\arctan\frac{\sqrt{1-t^{2}}}{\left|t\right|}\right)$	$\sin\left(\frac{1}{2}\arctan\frac{\sqrt{1-t^{2}}}{\left|t\right|}\right)$	$-\cos\left(\frac{1}{2}\arctan\frac{\sqrt{1-t^{2}}}{\left|t\right|}\right)$	$\cos\left(\frac{1}{2}\arctan\frac{\sqrt{1-t^{2}}}{\left|t\right|}\right)$
t=0	$1/\sqrt{2}$	$1/\sqrt{2}$	$-1/\sqrt{2}$	$1/\sqrt{2}$
0<t<1	$\cos\left(\frac{1}{2}\arctan\frac{\sqrt{1-t^{2}}}{t}\right)$	$\cos\left(\frac{1}{2}\arctan\frac{\sqrt{1-t^{2}}}{t}\right)$	$-\sin\left(\frac{1}{2}\arctan\frac{\sqrt{1-t^{2}}}{t}\right)$	$\sin\left(\frac{1}{2}\arctan\frac{\sqrt{1-t^{2}}}{t}\right)$
t=1	1	1	0	0
1<t	$\sqrt{t-\sqrt{t^{2}-1}}$	$\sqrt{t+\sqrt{t^{2}-1}}$	0	0

The presented dependences are shown graphically in Figure 1.

## Appendix B. Expressions for the Stress Tensor Components

(1) Expressions for functions appeared in (24):

$$\Psi_{p}(y)=\frac{1}{s^{2}}\left(s^{2}a^{2}\left(1-|s|ay\right)\exp(-|s|ay)-\int_{0}^{\infty }\left(1+|s|a\eta \right)R^{xx}(y,\eta )\exp(-|s|a\eta )d\eta \right)$$

$$\Psi_{q}(y)=\frac{1}{s^{2}}\left(|s|a(2-|s|ay)\exp(-|s|ay)-\int_{0}^{\infty }\eta R^{xx}(y,\eta )\exp(-|s|a\eta )d\eta \right)$$

$$\Psi_{T}(y)=-\frac{1}{s^{2}}\left(\Theta^{xx}(y)+\int_{0}^{\infty }\Theta (\eta )R^{xx}(y,\eta )d\eta \right)$$

Here,

$$\Theta^{xx}(y)=s^{2}\bar{T}(y)+\frac{|s{|}^{3}}{4a^{3}}\int_{0}^{\infty }\bar{T}(\eta )(-\left\{\left(1+3|s|a\eta -|s|ay(1+2|s|a\eta )\right)\alpha_{2}^{0}\right.$$

$$\left.+\;a^{2}(5-3|s|a\eta \;-|s|ay(3-2|s|a\eta ))\alpha_{1}^{0}\right\}\exp(-|s|a(y+\eta ))$$

$$+\left\{\left(1-|s|a|y-\eta |\right)\alpha_{2}^{0}-a^{2}\left(3-|s|a|y-\eta |\right)\alpha_{1}^{0}\right\}\exp(-|s|a|y-\eta |))d\eta$$

$$R^{xx}(y,\eta )=\sum_{n=0}^{\infty }K_{n+1}^{xx}(y,\eta )$$

$$K_{1}^{xx}(y,\eta )=-\frac{a|s{|}^{3}}{2}(a_{1}-a^{2})(\left(5\;-|s|a\left(3\eta \;+y(3-2|s|a\eta )\right)\right)\exp(-|s|a(y+\eta ))$$

$$+\;(3\;-|s|a|y-\eta |)\exp(-|s|a|y-\eta |))+2s^{2}(a_{1}-a^{2})\delta (y-\eta )\exp(-|s|a|y-\eta |)$$

$$K_{n+1}^{xx}(y,\eta )=\int_{0}^{\infty }K_{1}^{xx}(y,\tau )K_{n}(\tau ,\eta )d\tau ,\;n=1,2,\ldots$$

and δ(y−η) is Dirac’s delta function.

(2) Expressions for functions appeared in (25):

$$\vartheta_{p}(y)=\frac{i}{s}\left(-s^{2}a^{2}y\exp(-|s|ay)+\int_{0}^{\infty }\left(1+|s|a\eta \right)R^{xy}(y,\eta )\exp(-|s|a\eta )d\eta \right)$$

$$\vartheta_{q}(y)=\frac{i}{s}\left((1-|s|ay)\exp(-|s|ay)+\int_{0}^{\infty }\eta R^{xy}(y,\eta )\exp(-|s|a\eta )d\eta \right)$$

$$\vartheta_{T}(y)=\frac{i}{s}\left(\Theta^{xy}(y)+\int_{0}^{\infty }\Theta (\eta )R^{xy}(y,\eta )d\eta \right)$$

Here,

$$\Theta^{xy}(y)={\left(\frac{s}{2a}\right)}^{2}\int_{0}^{\infty }\bar{T}(\eta )(\left\{|s|a\left(\eta \;-y(1+2|s|a\eta )\right)\alpha_{2}^{0}\right.$$

$$\left.+\;a^{2}(2-|s|a\eta \;-|s|ay(3-2|s|a\eta ))\alpha_{1}^{0}\right\}\exp(-|s|a(y+\eta ))$$

$$+\left\{|s|a(y-\eta )\alpha_{2}^{0}+a^{2}\left(2\mathrm{sgn}(y-\eta )-|s|a(y-\eta )\right)\alpha_{1}^{0}\right\}\exp(-|s|a|y-\eta |))d\eta$$

$$R^{xy}(y,\eta )=\sum_{n=0}^{\infty }K_{n+1}^{xy}(y,\eta )$$

$$K_{1}^{xy}(y,\eta )=\frac{s^{2}}{2}(a_{1}-a^{2})((2\;-|s|a(\eta \;+y(3-2|s|a\eta )))\exp(-|s|a(y+\eta ))$$

+ (2sgn(y−η)−|s|a(y−η))exp(−|s|a|y−η|))

$$K_{n+1}^{xy}(y,\eta )=\int_{0}^{\infty }K_{1}^{xy}(y,\tau )K_{n}(\tau ,\eta )d\tau ,\;n=1,2,\ldots$$
